# Supplementary material for: A Critical View of the Application of the APEX Software (Aqueous Photochemistry of Environmentally-Occurring Xenobiotics) to Predict Photoreaction Kinetics in Surface Freshwaters
Source: Molecules. 2019 Dec 18;25(1):9. doi: 10.3390/molecules25010009 (PMC7017383; doi:10.3390/molecules25010009)
Supplement: Supplementary file 1 [file molecules-25-00009-s001.zip › 3.2.4_gcc-4.4.0.zip!/3.2.4_gcc-4.4.0/3.2.4_gcc-4.4.0/doc/octave/html/interpreter/Linear-Programming.html]

Linear Programming - Untitled


Next: Quadratic Programming,
Up: Optimization


---

### 24.1 Linear Programming

Octave can solve Linear Programming problems using the `glpk`
function. That is, Octave can solve

```
     min C'*x
```

subject to the linear constraints
A\*x = b where x >= 0.

The `glpk` function also supports variations of this problem.

— Function File: [xopt, fmin, status, extra] = **glpk** (c, a, b, lb, ub, ctype, vartype, sense, param)  
> Solve a linear program using the GNU GLPK library. Given three
> arguments, `glpk` solves the following standard LP:
>
> ```
>           min C'*x
> ```
>
> subject to
>
> ```
>           A*x  = b
>             x >= 0
> ```
>
> but may also solve problems of the form
>
> ```
>           [ min | max ] C'*x
> ```
>
> subject to
>
> ```
>           A*x [ "=" | "<=" | ">=" ] b
>             x >= LB
>             x <= UB
> ```
>
> Input arguments:
>
> c: A column array containing the objective function coefficients. a: A matrix containing the constraints coefficients. b: A column array containing the right-hand side value for each constraint in the constraint matrix. lb: An array containing the lower bound on each of the variables. If lb is not supplied, the default lower bound for the variables is zero. ub: An array containing the upper bound on each of the variables. If ub is not supplied, the default upper bound is assumed to be infinite. ctype: An array of characters containing the sense of each constraint in the constraint matrix. Each element of the array may be one of the following values `"F"`: A free (unbounded) constraint (the constraint is ignored). `"U"`: An inequality constraint with an upper bound (`A(i,:)*x <= b(i)`). `"S"`: An equality constraint (`A(i,:)*x = b(i)`). `"L"`: An inequality with a lower bound (`A(i,:)*x >= b(i)`). `"D"`: An inequality constraint with both upper and lower bounds (`A(i,:)*x >= -b(i)` *and* (`A(i,:)*x <= b(i)`). vartype: A column array containing the types of the variables. `"C"`: A continuous variable. `"I"`: An integer variable. sense: If sense is 1, the problem is a minimization. If sense is -1, the problem is a maximization. The default value is 1. param: A structure containing the following parameters used to define the behavior of solver. Missing elements in the structure take on default values, so you only need to set the elements that you wish to change from the default. Integer parameters: `msglev (LPX_K_MSGLEV, default: 1)`: Level of messages output by solver routines: 0: No output. 1: Error messages only. 2: Normal output . 3: Full output (includes informational messages). `scale (LPX_K_SCALE, default: 1)`: Scaling option: 0: No scaling. 1: Equilibration scaling. 2: Geometric mean scaling, then equilibration scaling. `dual (LPX_K_DUAL, default: 0)`: Dual simplex option: 0: Do not use the dual simplex. 1: If initial basic solution is dual feasible, use the dual simplex. `price (LPX_K_PRICE, default: 1)`: Pricing option (for both primal and dual simplex): 0: Textbook pricing. 1: Steepest edge pricing. `round (LPX_K_ROUND, default: 0)`: Solution rounding option: 0: Report all primal and dual values "as is". 1: Replace tiny primal and dual values by exact zero. `itlim (LPX_K_ITLIM, default: -1)`: Simplex iterations limit. If this value is positive, it is decreased by one each time when one simplex iteration has been performed, and reaching zero value signals the solver to stop the search. Negative value means no iterations limit. `itcnt (LPX_K_OUTFRQ, default: 200)`: Output frequency, in iterations. This parameter specifies how frequently the solver sends information about the solution to the standard output. `branch (LPX_K_BRANCH, default: 2)`: Branching heuristic option (for MIP only): 0: Branch on the first variable. 1: Branch on the last variable. 2: Branch using a heuristic by Driebeck and Tomlin. `btrack (LPX_K_BTRACK, default: 2)`: Backtracking heuristic option (for MIP only): 0: Depth first search. 1: Breadth first search. 2: Backtrack using the best projection heuristic. `presol (LPX_K_PRESOL, default: 1)`: If this flag is set, the routine lpx\_simplex solves the problem using the built-in LP presolver. Otherwise the LP presolver is not used. `lpsolver (default: 1)`: Select which solver to use. If the problem is a MIP problem this flag will be ignored. 1: Revised simplex method. 2: Interior point method. `save (default: 0)`: If this parameter is nonzero, save a copy of the problem in CPLEX LP format to the file "outpb.lp". There is currently no way to change the name of the output file. Real parameters: `relax (LPX_K_RELAX, default: 0.07)`: Relaxation parameter used in the ratio test. If it is zero, the textbook ratio test is used. If it is non-zero (should be positive), Harris' two-pass ratio test is used. In the latter case on the first pass of the ratio test basic variables (in the case of primal simplex) or reduced costs of non-basic variables (in the case of dual simplex) are allowed to slightly violate their bounds, but not more than `relax*tolbnd` or `relax*toldj (thus, relax is a percentage of tolbnd or toldj`. `tolbnd (LPX_K_TOLBND, default: 10e-7)`: Relative tolerance used to check if the current basic solution is primal feasible. It is not recommended that you change this parameter unless you have a detailed understanding of its purpose. `toldj (LPX_K_TOLDJ, default: 10e-7)`: Absolute tolerance used to check if the current basic solution is dual feasible. It is not recommended that you change this parameter unless you have a detailed understanding of its purpose. `tolpiv (LPX_K_TOLPIV, default: 10e-9)`: Relative tolerance used to choose eligible pivotal elements of the simplex table. It is not recommended that you change this parameter unless you have a detailed understanding of its purpose. `objll (LPX_K_OBJLL, default: -DBL_MAX)`: Lower limit of the objective function. If on the phase II the objective function reaches this limit and continues decreasing, the solver stops the search. This parameter is used in the dual simplex method only. `objul (LPX_K_OBJUL, default: +DBL_MAX)`: Upper limit of the objective function. If on the phase II the objective function reaches this limit and continues increasing, the solver stops the search. This parameter is used in the dual simplex only. `tmlim (LPX_K_TMLIM, default: -1.0)`: Searching time limit, in seconds. If this value is positive, it is decreased each time when one simplex iteration has been performed by the amount of time spent for the iteration, and reaching zero value signals the solver to stop the search. Negative value means no time limit. `outdly (LPX_K_OUTDLY, default: 0.0)`: Output delay, in seconds. This parameter specifies how long the solver should delay sending information about the solution to the standard output. Non-positive value means no delay. `tolint (LPX_K_TOLINT, default: 10e-5)`: Relative tolerance used to check if the current basic solution is integer feasible. It is not recommended that you change this parameter unless you have a detailed understanding of its purpose. `tolobj (LPX_K_TOLOBJ, default: 10e-7)`: Relative tolerance used to check if the value of the objective function is not better than in the best known integer feasible solution. It is not recommended that you change this parameter unless you have a detailed understanding of its purpose.
>
> Output values:
>
> xopt: The optimizer (the value of the decision variables at the optimum). fopt: The optimum value of the objective function. status: Status of the optimization. Simplex Method: 180 (`LPX_OPT`): Solution is optimal. 181 (`LPX_FEAS`): Solution is feasible. 182 (`LPX_INFEAS`): Solution is infeasible. 183 (`LPX_NOFEAS`): Problem has no feasible solution. 184 (`LPX_UNBND`): Problem has no unbounded solution. 185 (`LPX_UNDEF`): Solution status is undefined. Interior Point Method: 150 (`LPX_T_UNDEF`): The interior point method is undefined. 151 (`LPX_T_OPT`): The interior point method is optimal. Mixed Integer Method: 170 (`LPX_I_UNDEF`): The status is undefined. 171 (`LPX_I_OPT`): The solution is integer optimal. 172 (`LPX_I_FEAS`): Solution integer feasible but its optimality has not been proven 173 (`LPX_I_NOFEAS`): No integer feasible solution. If an error occurs, status will contain one of the following codes: 204 (`LPX_E_FAULT`): Unable to start the search. 205 (`LPX_E_OBJLL`): Objective function lower limit reached. 206 (`LPX_E_OBJUL`): Objective function upper limit reached. 207 (`LPX_E_ITLIM`): Iterations limit exhausted. 208 (`LPX_E_TMLIM`): Time limit exhausted. 209 (`LPX_E_NOFEAS`): No feasible solution. 210 (`LPX_E_INSTAB`): Numerical instability. 211 (`LPX_E_SING`): Problems with basis matrix. 212 (`LPX_E_NOCONV`): No convergence (interior). 213 (`LPX_E_NOPFS`): No primal feasible solution (LP presolver). 214 (`LPX_E_NODFS`): No dual feasible solution (LP presolver). extra: A data structure containing the following fields: `lambda`: Dual variables. `redcosts`: Reduced Costs. `time`: Time (in seconds) used for solving LP/MIP problem. `mem`: Memory (in bytes) used for solving LP/MIP problem (this is not available if the version of GLPK is 4.15 or later).
>
> Example:
>
> ```
>           c = [10, 6, 4]';
>           a = [ 1, 1, 1;
>                10, 4, 5;
>                 2, 2, 6];
>           b = [100, 600, 300]';
>           lb = [0, 0, 0]';
>           ub = [];
>           ctype = "UUU";
>           vartype = "CCC";
>           s = -1;
>           
>           param.msglev = 1;
>           param.itlim = 100;
>           
>           [xmin, fmin, status, extra] = ...
>              glpk (c, a, b, lb, ub, ctype, vartype, s, param);
> ```
